# Ankylosing Spondylitis Patients Have Impaired Osteoclast Gene Expression in Circulating Osteoclast Precursors

**DOI:** 10.3389/fmed.2017.00005

**Published:** 2017-01-27

**Authors:** Inês P. Perpétuo, Joana Caetano-Lopes, Elsa Vieira-Sousa, Raquel Campanilho-Marques, Cristina Ponte, Nikita Khmelinskii, Helena Canhão, Mari Ainola, João E. Fonseca

**Affiliations:** ^1^Rheumatology Research Unit, Faculdade de Medicina, Instituto de Medicina Molecular, Universidade de Lisboa, Lisboa, Portugal; ^2^Rheumatology Department, Hospital de Santa Maria, Centro Hospitalar Lisboa Norte, EPE, Lisbon Academic Medical Centre, Lisboa, Portugal; ^3^EpiDoC Unit, Chronic Diseases Research Center (CEDOC), NOVA Medical School, Universidade Nova de Lisboa, Lisboa, Portugal; ^4^Musculoskeletal Diseases and Inflammation Research Group, Biomedicum Helsinki 1, Faculty of Medicine, Institute of Clinical Medicine, University of Helsinki, Helsinki, Finland

**Keywords:** monocytes, osteoclasts, ankylosing spondylitis, CSF1R, RANK, NFATc1, gene expression, humans

## Abstract

**Introduction:**

Ankylosing spondylitis (AS) is typically characterized by focal bone overgrowth and also by systemic bone loss. We hypothesize that the increased osteoproliferation found in AS might be partially due to reduced ability of osteoclast precursors (OCPs) to differentiate into osteoclasts (OCs). Therefore, our aim was to characterize bone remodeling and pro-osteoclastogenesis inflammatory environment, monocytes’ phenotype, and *in vitro* osteoclast differentiation in AS patients.

**Methods:**

Patients with active AS without any ongoing therapy and age- and gender-matched healthy donors were recruited. Receptor activator of nuclear factor-κβ (RANKL) surface expression on circulating leukocytes and frequency and phenotype of monocyte subpopulations were assessed. Quantification of serum levels of bone turnover markers and cytokines, *in vitro* OC differentiation assay and quantitative reverse transcription real-time PCR for OC-specific genes were performed.

**Results:**

Pro- and anti-inflammatory cytokine serum levels were higher in AS patients than in controls. RANKL neutrophil expression was higher in AS patients when compared to healthy donors, but CD51/CD61 expression was lower in the classical monocyte subpopulation. Concerning osteoclastogenesis, we found no differences in the *in vitro* osteoclast differentiating potential of these cells when compared to healthy donors. However, we observed low expression of CSF1R, RANK, and NFATc1 in AS OCPs.

**Conclusion:**

Despite the high levels of pro-inflammatory cytokines present in AS patients, no differences in the number of OC or resorbed area were found between AS patients and healthy donors. Moreover, we observed that OCPs have low OC-specific gene expression. These findings support our hypothesis of an impaired response of OCPs to pro-osteoclastogenic stimuli *in vivo* in AS patients.

## Introduction

Ankylosing spondylitis (AS) is an immune-mediated chronic inflammatory disease, affecting predominantly the axial skeleton and enthesis. Although its etiopathology is still unknown, tumor necrosis factor (TNF) and interleukin (IL)-17A have been shown to play a central role (1, 2). In AS, chronic inflammation has a systemic impact on bone, which is reflected by a higher incidence of osteoporosis and increased bone fragility, although spine BMD assessment is difficult due to the presence of syndesmophytes (3). In fact, in contrast with RA, trabecular bone loss in AS is accompanied by new bone formation at the enthesis sites (4, 5).

The immune and skeletal systems have several regulatory factors in common and immune system cells have a profound influence on bone metabolism, particularly in chronic inflammatory diseases. Monocytes, the circulating osteoclast precursors (OCPs) cells, are phenotypical and functionally heterogeneous and play a critical regulatory role in inflammation and innate immune responses (6, 7). Monocytes can be divided into three subpopulations based on their expression of CD14 and CD16 surface markers. The classical subset CD14^bright^CD16^−^ comprises the phagocytic monocytes, the non-classical subset CD14^dim^CD16^+^ is responsible for cytokine production and T cell activation and the most recently described intermediate subset, CD14^bright^CD16^+^, which comprises specialized antigen-presenting cells (7). Although monocytes from the classical subpopulation are considered OCPs, all of these subpopulations can differentiate into osteoclasts (OCs) (8, 9).

Receptor activator of nuclear factor κB (RANK) ligand (10) is a key molecule for OC differentiation and activity. Receptor activator of nuclear factor-κβ (RANKL) is present on osteoblasts surface and is also expressed by activated immune cells. These cells contribute indirectly to OC differentiation and function through the secretion of pro-inflammatory cytokines, particularly TNF, IL-1β, IL-6, and IL-17A (2, 11, 12). Taken together, this environment contributes to increased OC differentiation and, consequently, bone resorption (11, 12).

It has been previously postulated that AS may have an early phase, featured by erosions in sacroiliac joints and enthesis, and a later stage, when the disease course is dominated by new bone formation (syndesmophytes and ankylosis) (4). Therefore, AS is characterized by exaggerated repair and bone overgrowth (13). Evidence from animal models suggests that dickkopf-related protein (DKK)-1 and sclerostin (SOST), both inhibitors of the Wnt/β-catenin pathway and thus inhibitors of osteoblast function, are downregulated in AS, resulting in increased osteogenesis (14, 15). On the other hand, previous studies have found subtle histological differences in the synovial tissue between AS and RA patients, namely the RANKL/osteoprotegerin (OPG) ratio, which was shown to be significantly lower in AS (16). However, not much attention has been given to the bone resorption features of AS. We have previously shown in a cohort of AS patients before TNF-blocking therapy decreased *in vitro* differentiation rate of monocytes into OC (17).

In this study, we hypothesize that AS OCP have impaired capacity to respond to osteoclastogenic stimuli, irrespective of inflammation in AS. Therefore, the aim of this study was to characterize bone remodeling and pro-osteoclastogenesis inflammatory environment, monocytes’ phenotype, and *in vitro* OC differentiation in AS patients.

## Patients and Methods

### Patients

Patients who fulfilled the 1984 New York modified criteria for AS (18) were recruited from the Rheumatology and Bone Metabolic Disease Department, Hospital de Santa Maria, Lisbon Academic Medical Centre, Portugal. Inclusion criteria included active disease, defined as an AS disease activity score (ASDAS-CRP) > 1.3 (19) and documented axial involvement by X-ray or magnetic resonance imaging (20). Patients previously exposed or presently under disease-modifying anti-rheumatic drugs (DMARDs) or biological DMARDs were excluded. The previous use of non-steroidal anti-inflammatory drugs was allowed. Information about gender, age, disease duration, HLA-B27 status, peripheral manifestations, and the presence of syndesmophytes was collected. Erythrocyte sedimentation rate and C-reactive protein (CRP) were measured and the ASDAS, the Bath Ankylosing Spondylitis Disease Activity Index (BASDAI) (21), and the Bath Ankylosing Spondylitis Functional Index (BASFI) (22) were assessed. Twenty-one healthy donors matched for age and gender were used as controls. The controls were recruited from the research institute and hospital staff population and exclusion criteria included self-reported auto-immune diseases like asthma and axial or joint pain/condition.

Heparinized blood from each participant was used for whole blood flow cytometry and peripheral blood mononuclear cell (PBMC) isolation. The Hospital de Santa Maria ethics committee approved this study, and all participants signed an informed consent. Patient’s management was performed in accordance with the standard practice, and this study was conducted in accordance with the Declaration of Helsinki, as amended in Brazil (2013).

### Cytokine Detection in the Serum

Interleukin-1β, IL-2, IL-4, IL-6, IL-8, IL-10, IL-12p70, IL-17A, IL-18, IL-22, interferon gamma (IFN-γ), macrophage inflammatory protein (MIP-1α), transforming growth factor-beta (TGF-β), and TNF levels were measured in the serum by FlowCytomix custom assay kits (Bender MedSystems), according to the manufacturer’s instructions. Samples were acquired using a FACSCalibur flow cytometer (BD Biosciences). Raw data of the flow cytometry bead assay were analyzed using the FlowCytomix Pro 3.0 software (Bender MedSystems). Carboxy-terminal type I collagen crosslinks (CTX-I; Sunred Biological Technology), human type I procollagen amino-terminal-propeptide (P1NP; Sunred Biological Technology), OPG, SOST, DKK-1, and soluble RANKL (Biomedica Gruppe) were quantified by ELISA according to the manufacturer’s instructions.

### Antibodies and Flow Cytometry

Identification of B and T cells and neutrophils in peripheral blood, and immunophenotyping of monocytes in the PBMC samples, were performed using matched combinations of anti-human murine mAbs. For peripheral blood staining, anti-CD19 PerCP-Cy5.5 (eBioscience), anti-CD3 PerCP (BD Biosciences), anti-CD66b FITC (Immunotools), and anti-RANKL PE (Santa Cruz Biotechnology) were used. Monocyte subpopulations were identified with anti-CD14 FITC (BD Biosciences) or PerCPCy5.5 (Immunotools) and anti-CD16 APC (Immunotools) and stained with combinations of anti-CD11b PE-Cy7, CD105 PE, CD62L PE-Cy7, CD51/CD61 FITC (eBioscience), CCR2 PE (R&D Systems), HLA-DR PerCP (BD Biosciences), and RANK PE (Santa Cruz Biotechnology). Cell death was assessed by staining with Annexin V Apoptosis Detection Kit APC (eBioscience). Acquisition was performed using a FACSCalibur (BD Biosciences). Heparinized whole blood was used for staining. Erythrocytes were lysed with red blood cell lysis buffer, and cells were incubated with IgG block solution 300 ng/mL (ChromPure Mouse IgG whole molecule; Jackson ImmunoResearch Laboratories) before staining. Absolute cell counts were calculated from differential leukocyte count determined for all participants. PBMCs were isolated by density-gradient centrifugation with Histopaque^®^-1077 (Sigma-Aldrich). Monocyte subpopulations were identified as described before based on their CD14 and CD16 surface expression (7). Data were analyzed using the FlowJo software (Tree Star Inc., Stanford University).

### Osteoclast Differentiation

Peripheral blood mononuclear cells isolated by density-gradient centrifugation were plated in 96-well culture plates at a density of 7.0 × 10^5^ cells/well. PBMCs were left overnight for OC precursors to adhere on bone slices and were further cultured for 21 days with M-CSF (25 ng/mL), sRANKL (50 ng/mL), dexamethasone (10 nM), and TGF-β (2.5 ng/mL), as described previously (17, 23, 24). The culture medium was then changed twice a week. Cells cultured on bone slices for 7, 14, and 21 days (23, 25) were used for functional assays and gene expression.

### Functional Assays

TRAP staining of OCs was performed using the Acid Phosphate, Leukocyte Kit (TRAP; Sigma-Aldrich) according to the manufacturer’s instructions for counting mature OCs (TRAP-positive cells containing three or more nuclei). In the resorption assay, to measure the resorbed area, OCs were removed from the bone slices using sodium hypochlorite and stained with 0.1% toluidine blue. Bone slices from both TRAP staining and resorption functional assays were photographed in an area of 1.25 mm^2^ using a bright field microscope (Leica DM2500; Leica). The number of TRAP stained OCs was counted for each time point, per condition, and the resorption pits were traced using the ImageJ (NIH, Bethesda, MD, USA). The resorbed area was calculated and expressed in percentage of total area.

### Gene Expression

RNA was extracted from cells at days 1, 7, 14, and 21 of culture using NZYol (NZYTech). Following RNA extraction, total RNA concentration and purity was quantified using Nanodrop 1000 (Thermo Scientific). Complementary (c)DNA was synthesized at a concentration of 0.6 ng/µL using the DyNAmo™ cDNA Synthesis Kit (Thermo Scientific) according to the manufacturer’s instructions. Genes that encode for osteoclast proteins such as CSF1R, RANK, NFATc1, ATP6V0D2, and CTSK were studied (see Table S1 in Supplementary Material for primers). Ribosomal RNA 18s was chosen as the housekeeping gene. Primers were designed using the primer-BLAST (26) software, and qPCR was performed using the DyNAmo™ Flash SYBR Green qPCR Kit (Thermo Scientific). The efficiency of qPCR was analyzed using the standard curve method, as described previously (27, 28). The values obtained were normalized with the housekeeping gene 18s rRNA.

### Statistical Analysis

Statistical analysis was performed using the SPSS Statistics 17.0 (IBM, USA). Categorical variables were expressed as frequencies and differences were tested using chi-square test. Continuous variables were expressed by median and interquartile range. Medians were compared using the Mann–Whitney test while correlation between two variables was tested using Spearman correlation. The *p*-values less than 0.05 were considered statistically significant.

## Results

### Population Characteristics

Thirty AS patients, 11 women and 19 men, with a median age of 40 [33–51] years and ASDAS-CRP of 3.8 [2.4–4.0] were enrolled in this study as well as 21 healthy donors, 8 women and 13 men, with a median age of 45 [36–53] years. All patients presented axial involvement documented by X-ray or MRI; however, only 30% of the patients presented syndesmophyte formation. The characteristics of each group are summarized in Table 1.

**Table 1:** **Characterization of the studied population**.

	AS patients (*n* = 30)	Healthy (*n* = 21)	*p*-Value
Age (years)	40 [33–51]	45 [36–53]	0.4081
Male (%)	63%	62%	1.0000
Symptoms duration (years)	11 [8–21]	NA	
ESR (mm/h)	23 [11–38]	NA	
CRP (mg/dL)	0.8 [0.2–1.7]	NA	
ASDAS-CRP	3.8 [2.4–4]	NA	
BASDAI	5.8 [3.7–7.3]	NA	
BASFI	5.7 [2.1–7.5]	NA	
HLA-B27 (% positive)	77%	NA	
Presence of syndesmophytes (%)	30%	NA	
Peripheral involvement (%)	37%	NA	
Treatment with NSAIDs (%)	60%	NA	
NSAIDs duration (months)	12 [6–52]	NA	

*Data are represented as median [interquartile range] unless stated otherwise*.

*NA, not applicable; AS, ankylosing spondylitis; ESR, erythrocyte sedimentation rate; CRP, C-reactive protein; ASDAS, Ankylosing Spondylitis Disease Activity Score; BASDAI, Bath Ankylosing Spondylitis Disease Activity Index; BASFI, Bath Ankylosing Spondylitis Functional Index; HLA, human leukocyte antigen; NSAIDs, non-steroidal anti-inflammatory drugs*.

### Pro-inflammatory Cytokine Levels Were Higher in AS Patients than in Controls

All the analyzed cytokines and chemokines had significantly higher circulating levels in AS patients when compared to healthy donors (IL-1β, *p* = 0.0001; IL-2, *p* = 0.0005; IL-4, *p* = 0.0211; IL-6, *p* = 0.0005; IL-8, *p* = 0.0001; IL-10, *p* = 0.0353; IL-12p70, *p* = 0.00189; IL-17A, *p* = 0.0010; IL-18, *p* = 0.0002; IL-22, *p* = 0.0332; IFN-γ, *p* = 0.0005; MIP-1α, *p* = 0.0428; TGF-β, *p* = 0.0003; TNF, *p* = 0.0189; Table S2 in Supplementary Material). After adjusting for multiple comparisons only IL-1β, IL-2, IL-6, IL-8, IL-17A, IL-18, IFN-γ, TGF-β, and TNF remained significantly higher in AS patients as compared to controls. No correlation was found between cytokine levels and disease activity scores (BASDAI or ASDAS). Positive correlations were found between the functional activity index BASFI and the circulating levels of IL-1β (*r* = 0.414, *p* = 0.004), IL-6 (*r* = 0.485, *p* = 0.014), IL-8 (*r* = 0.407, *p* = 0.019), IL-10 (*r* = 0.498, *p* = 0.011), IL17A (*r* = 0.489, *p* = 0.013), and IL-18 (*r* = 0.415, *p* = 0.039) in AS patients.

### Systemic Bone Turnover Markers Were Lower in AS Patients As Compared to Healthy Controls

**Figure 1:**
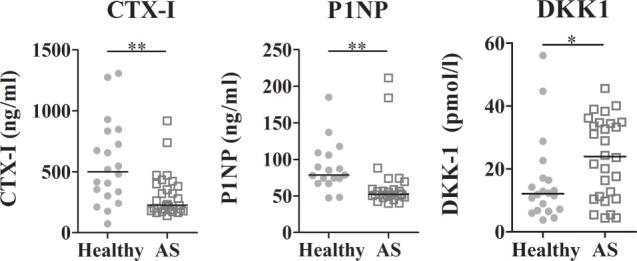
**Serum levels of bone turnover markers and DKK1**. CTX-I and P1NP were decreased in AS patients when compared to healthy controls. DKK1 circulating levels were higher in AS patients than in healthy controls. Each dot represents a sample. Line represents median (*p < 0.05 and **p < 0.01). CTX-I, carboxy-terminal telopeptide of type I collagen; P1NP, total procollagen type 1 N-terminal propeptide; DKK1, dickkopf-related protein 1.

Regarding the bone remodeling proteins, no differences in circulating levels of sRANKL, OPG, and SOST were found, but DKK-1 was significantly higher in AS patients when compared to healthy donors (*p* = 0.0394; Table S2 in Supplementary Material; Figure 1). The levels of CTX-I and P1NP were lower in AS patients when compared to healthy donors (Figure 1), but no difference in the CTX-I/P1NP ratio was found. After adjusting for multiple comparisons, P1NP and CTX-I remained significantly lower in AS patients. No association was found between bone turnover markers and disease activity scores.

### RANKL Neutrophil Expression Was Higher in AS Patients When Compared to Healthy Controls

**Figure 2:**
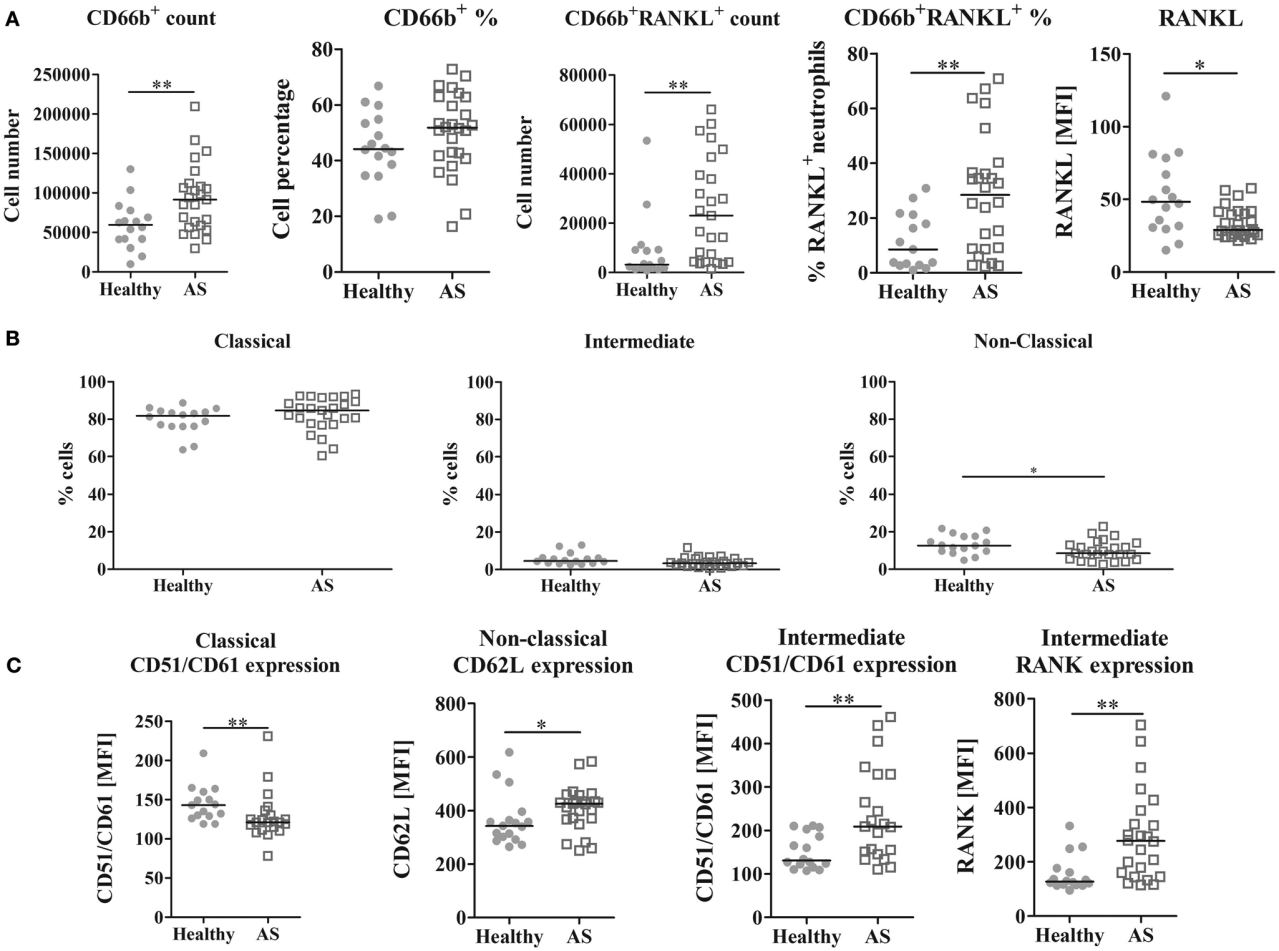
**Leukocyte analysis by flow cytometry**. **(A)** CD66b^+^ circulating cells are increased in AS patients when compared to healthy donors (*p* = 0.0059 for absolute number count). CD66b^+^RANKL^+^ cells are increased in the circulation of AS patients when compared to healthy donors (both in absolute number and in percentage, *p* = 0.0022 and 0.0071, respectively). However, RANKL expression on neutrophils surface is significantly decreased in AS patients when compared to healthy donors (*p* = 0.0134). No differences were observed for T or B lymphocytes in circulation. **(B)** The frequency of the non-classical monocyte subpopulation (CD14^dim^CD16^+^) is decreased in AS patients when compared to healthy donors (*p* = 0.0490). No differences were found in the circulating classical or intermediate subpopulations. **(C)** Phenotype of circulating monocyte subpopulations; CD51/CD61 (αVβ3 integrin) is decreased in the classical subpopulation in AS patients (*p* = 0.0074); however, it is increased in the intermediate subpopulation of AS patients when compared to healthy donors (*p* = 0.0061); CD62L (L-selectin) is increased in the non-classical subpopulation of AS patients when compared to healthy donors (*p* = 0.0457) and RANK expression is increased in the intermediate subpopulation of AS patients when compared to healthy donors (*p* = 0.0011). Each dot represents a sample. Line represents median. *p* < 0.05 is considered significant (*p < 0.05 and **p < 0.01). AS, ankylosing spondylitis; MFI, median fluorescence intensity; RANK (L), receptor activator of nuclear factor-κβ (ligand).

The frequency of neutrophils, B, and T lymphocytes was analyzed, as well as RANKL surface expression in these cells. The number of neutrophils was higher in AS patients when compared to the control group (*p* = 0.0059; Figure 2A). Regarding RANKL surface expression, a higher number and percentage of RANKL-expressing neutrophils in AS patients were found when compared to healthy donors (*p* = 0.0022 and 0.0071, respectively); however, surface RANKL was lower on these cells (*p* = 0.0134) when compared to controls. No differences were observed in the total circulating T or B lymphocytes (percentage and total number), in RANKL surface expression or in cell death between any of the studied populations.

### CD51/CD61 (αvβ3 Integrin) Surface Expression Was Lower in OCPs from AS Patients

Monocyte subpopulations were analyzed after PBMC isolation and staining. The frequency of non-classical CD14^dim^CD16^+^ monocytes was lower in AS patients when compared to control donors (*p* = 0.0490; Figure 2B). No differences were found between groups in the intermediate CD14^bright^CD16^+^ or in the classical CD14^brigth^CD16^−^ (data not shown) subpopulations. Spearman correlation showed that, in AS patients, the frequency of classical and non-classical subpopulations was inversely correlated (*r* = −0.948, *p* < 0.001).

Regarding the phenotype of circulating monocytes, in AS patients, the classical subpopulation had lower surface expression of CD51/CD61 (αvβ3 integrin) (*p* = 0.0074; Figure 2C). The intermediate subpopulation had a higher expression of CD51/CD61 and RANK (*p* = 0.0061 and 0.0011, respectively), and the non-classical subpopulation had more CD62L surface expression (*p* = 0.0457), when compared to healthy controls. No differences in cell death between groups were found (data not shown).

### Osteoclast Differentiating Genes Were Downregulated in OCPs from AS Patients

**Figure 3:**
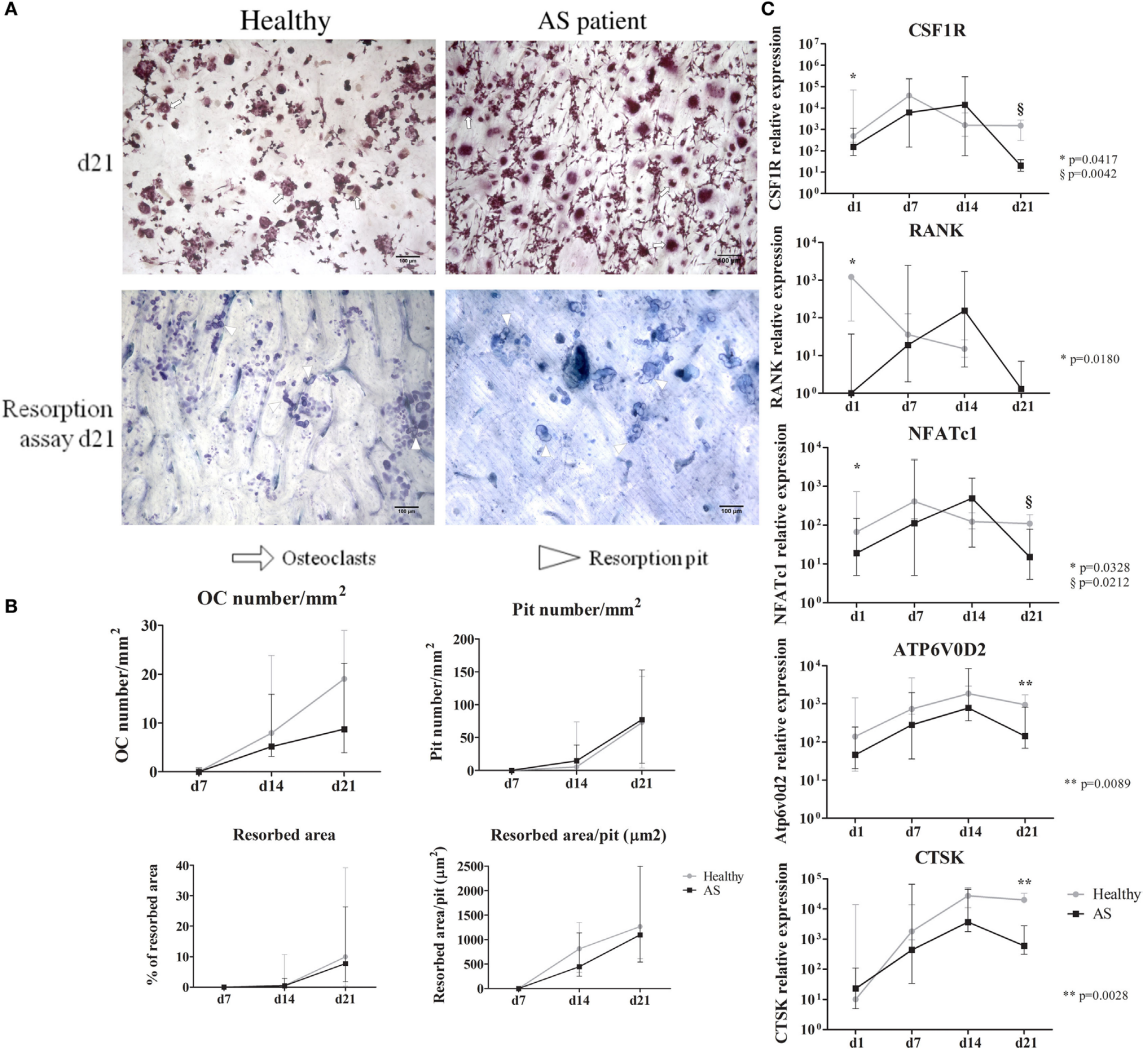
**Functional assays and osteoclast gene expression**. **(A)** Representative images of TRAP staining and resorption pits at culture day 21 are shown for healthy and AS patients. (10× magnification). White arrows—osteoclasts; white arrowheads—resorption pit. **(B)** Osteoclast number, resorbed area, pit number, and resorbed area/pit increased throughout time without significant differences between groups. **(C)** Gene expression profile of cells in culture for 21 days. CSF1R, RANK, and NFATc1 are significantly decreased at day 1 in cells from AS patients when compared to healthy donors. We found no differences between groups throughout the culture time except at culture day 21 where all genes are significantly higher in cells differentiated from healthy donors except for RANK. Relative gene expression shown in Log scale. Dots in graphs represent median gene expression for each group at each time-point and bars represent interquartile range. d, day; OC, osteoclast; CSF1R, gene encoding macrophage-colony stimulating factor (c-fms); RANK, gene encoding for receptor activator of nuclear factor-κβ; NFATc1, gene encoding for nuclear factor of activated T-cells; Atp6v0d2, gene encoding ATPase, H^+^ transporting, lysosomal V0 subunit D2; CTSK, gene encoding cathepsin K. *p* < 0.05 is considered significant.

Osteoclast number increased from culture days 7 to 21. Although differences were not found between groups, at culture day 21, AS patients had less OCs than healthy donors. The number of nuclei per OC was similar between groups. Pit number and percentage of resorption area markedly increased after day 14 in both groups; however, no differences in the resorption activity of these cells or in the pit size were found between groups (Figure 3A).

Regarding OC gene expression, CSF1R, RANK, and NFATc1 were significantly downregulated at day 1 in AS patients, when compared to healthy donors (Figure 3B). On the last culture day, CSF1R, NFATc1, ATP6V0D2, and CTSK expression was significantly reduced in cells from AS patients. After correcting for multiple comparisons, ATP6V0D2 and CTSK expression at culture day 21 remained significantly reduced in cells from AS patients.

### Subanalysis of the Group of Patients under 45 Years of Age, HLA-B27-Positive, and with Syndesmophytes

We have also performed a subanalysis of the group of patients under 45 years of age, HLA-B27-positive, and with syndesmophytes and compared them with age-matched controls in order to explore our results in a more homogenous population. Six patients met these criteria: three females and one male with a median age of 38 [34–42] years. These patients had median disease duration of 12 [11–22] years, median ASDAS of 4.1 [2.3–4.6], and median BASDAI of 7.1 [5.4–7.6]. Ten controls were under 45 years of age, one female and nine males with a median age of 34 [30–36] years. This subanalysis showed that only IL-22 remained significantly increased in this subgroup of AS patients when compared to controls (*p* = 0.0347). CTX and P1NP levels were decreased in this group of patients when compared to the controls (*p* = 0.0110 and 0.0007, respectively). The absolute number of RANKL^+^ neutrophils was increased when compared to controls and the RANKL MFI was reduced in this subpopulation (*p* = 0.0196 and 0.0336, respectively). The monocyte non-classical subpopulation remained decreased in the AS subpopulation when compared to the healthy controls (*p* = 0.0498), and no further differences in the phenotype of the monocyte subpopulations were found in the subanalysis. No differences were found in the number of OC or of resorption pit per square millimeter. Unlike observed in the total subpopulation, we found in this subanalysis that at day 21 the percentage of area resorbed by cells from the AS patients subpopulation was significantly lower than the area resorbed by the controls (*p* = 0.0400). When analyzing gene expression in these differentiated cells, we found that both NFATc1 and the ATPase subunit expression were significantly lower in cells from the AS patients subpopulation when compared to the healthy controls (*p* = 0.0028 and 0.0056, respectively).

## Discussion

We hypothesized that AS OC precursors have impaired capacity to respond to osteoclastogenic stimuli, regardless of inflammation in AS. In this study, we characterized the bone remodeling and pro-osteoclastogenic inflammatory environment, monocytes’ phenotype, and *in vitro* OC differentiation of OCP in AS patients without any disease-modifying therapy.

Serum levels of IL-1β, IL-2, IL-6, IL-8, IL-17A, IL-18, IFN-γ, TGF-β, and TNF were significantly higher in AS patients when compared to controls, as described before (1, 29–32). As previously described, the majority of these cytokines are pro-inflammatory and pro-osteoclastogenic (1, 2, 8, 11, 30, 31, 33). Of interest, TGF-β plays an anti-inflammatory role and is also involved in wound healing and fibrosis (34), and it has been described as an important cytokine for bone formation (35).

Sclerostin and DKK1 are endogenous inhibitors of the Wnt/β-catenin pathway; these proteins are mainly secreted by osteocytes and inhibit osteoblasts differentiation (36). SOST serum levels were not significantly different between AS and controls, but DKK-1 was significantly higher. Studies assessing levels of DKK-1 and SOST in AS are scarce and have provided conflicting results (15, 33, 37). Taylan et al. described that serum levels of DKK-1 and SOST were not different in AS patients with mild to active disease when compared to healthy controls (29, 33). In contrast, other studies reported lower levels of circulating SOST and of DKK-1 in AS patients when compared to healthy donors. Our results are consistent with a study by Miceli-Richard and colleagues who found no differences between serum levels of SOST when comparing AS patients with healthy donors (37).

When looking at the RANKL/OPG axis, no differences in sRANKL, OPG, or sRANKL/OPG ratio were found between groups. Previous studies have shown discrepancies in sRANKL and OPG serum levels in AS patients. In some studies, sRANKL and OPG have been found to be increased in AS patients with active disease (38); however, in other studies, AS patients with mild to active disease have lower sRANKL/OPG (33, 39) than controls. Different results for sRANKL measurements might be due to different patient’s characteristics, sample size, and also to the use of different detection methods.

CTX-I and P1NP levels were lower in AS patients when compared to healthy controls, reflecting a low bone turnover. However, recent reports have shown increased levels of urinary CTX-I levels in AS patients when compared to healthy donors (40). Bone turnover markers values remain highly controversial in the literature, since they are also affected by time of collection and circadian rhythms (41, 42).

Circulating leukocyte numbers and RANKL expression was analyzed by flow cytometry. We showed that there was an increase in the total number of neutrophils as previously shown by several studies in AS patients (43–46). We also observed higher numbers of RANKL^+^ neutrophils, despite the lower surface expression of this protein, as compared to controls. It was previously shown that activated neutrophils express RANKL on their surface and are capable of promoting osteoclastogenesis in other rheumatic diseases and healthy donors (47, 48).

Monocytes can be classified into three subpopulations that are phenotypically and functionally heterogeneous. The classical subpopulation is the most abundant subset and includes the OCP. Although the function of monocytes is well characterized in inflammation, their role in AS axial disease is not clear (6, 7). CD51/CD61 (α_V_β_3_ integrin) is important for monocyte and OC adhesion to the bone matrix and for providing survival signals and mobility traits. According to our results, CD51/CD61 expression was lower in the classical subpopulation of AS patients and in the intermediate subpopulation both CD51/CD61 and RANK expression were higher, in comparison to controls. To the best of our knowledge, this is the first time that the phenotype of the previously described monocyte subpopulations (7) has been addressed in AS patients. The decreased CD51/CD61 expression in the classical monocyte subpopulation, which has traditionally been regarded as the OC precursor, may be a clue for the pattern of bone resorption seen in AS. This observation is consistent with the study performed by Gengenbacher and colleagues who showed lower bone resorption *in vitro* in AS when compared to RA patients, although the different monocytes’ subpopulations were not assessed (49).

No differences were found in the number of OCs, the number of resorption pits, or the resorbed area. However, OC-specific gene expression was significantly lower in cells from AS patients when compared to healthy donors. This was observed not only at culture day 1 for CSF1R, RANK, and NFATc1 but also at day 21 for CSF1R, NFATc1, ATP6V0D2, and CTSK. We believe that this decrease in OC-specific genes expression in the circulating precursors and the significant decrease of the expression of genes that encode bone degrading proteins at culture day 21 are indicators of reduced osteoclastogenesis in AS, probably due to poor response to osteoclastogenic stimuli.

High levels of pro-inflammatory cytokines and increased surface RANKL expression have been previously associated with increased OC differentiation and activity. According to our study, despite high levels of pro-inflammatory cytokines (IL-1β, IL-6, IL-17A, and TNF) and of high number of RANKL-expressing neutrophils, no differences were found in OC number or activity between AS patients and healthy controls. Moreover, OC-specific gene expression was significantly lower in cells from AS patients when compared to healthy donors. We believe that the decrease in OC-specific genes expression in the circulating precursors and the significant decrease of the expression of genes that encode bone-degrading proteins at culture day 21 are indicators of reduced osteoclastogenesis in AS.

We propose that the low RANK and CD51/CD61 expression in classical monocytes contribute to decrease OC formation and adhesion to bone, resulting in reduced capacity of these cells to resorb bone.

Although chronic inflammatory diseases like RA and psoriatic arthritis are classically associated with increased bone resorption (50–55), our data in AS patients (including or excluding HLA-B27-negative patients without syndesmophytes) show decreased indicators of OC formation and bone resorption, suggesting reduced capacity of OCPs to differentiate into OC and resorb bone.

## Author Contributions

Conceived and designed the experiments: IP, JC-L, HC, MA, and JF. Performed the experiments: IP and JC-L. Analyzed the data: IP, JC-L, RC-M, CP, NK, EV-S, and HC. Contributed reagents/materials/analysis tools: IP, RC-M, CP, EV-S, NK and HC. Wrote, reviewed, and accepted the final version of the paper: IP, JC-L, RC-M, CP, EV-S, NK, HC, MA, and JF.

## Conflict of Interest Statement

The authors declare that the research was conducted in the absence of any commercial or financial relationships that could be construed as a potential conflict of interest.
