# Corrigendum: Ankylosing Spondylitis Patients Have Impaired Osteoclast Gene Expression in Circulating Osteoclast Precursors

**DOI:** 10.3389/fmed.2017.00038

**Published:** 2017-04-12

**Authors:** Inês P. Perpétuo, Joana Caetano-Lopes, Elsa Vieira-Sousa, Raquel Campanilho-Marques, Cristina Ponte, Nikita Khmelinskii, Helena Canhão, Mari Ainola, João E. Fonseca

**Affiliations:** ^1^Rheumatology Research Unit, Faculdade de Medicina, Instituto de Medicina Molecular, Universidade de Lisboa, Lisboa, Portugal; ^2^Rheumatology Department, Hospital de Santa Maria, Centro Hospitalar Lisboa Norte, EPE, Lisbon Academic Medical Centre, Lisboa, Portugal; ^3^EpiDoC Unit, Chronic Diseases Research Center (CEDOC), NOVA Medical School, Universidade Nova de Lisboa, Lisboa, Portugal; ^4^Musculoskeletal Diseases and Inflammation Research Group, Biomedicum Helsinki 1, Faculty of Medicine, Institute of Clinical Medicine, University of Helsinki, Helsinki, Finland

**Keywords:** monocytes, osteoclasts, ankylosing spondylitis, CSF1R, RANK, NFATc1, gene expression, humans

In the original article, the name of the author Nikita Khmelinskii was missing by mistake. The authors apologize for this oversight.

**New Author Contribution Statement:**

Conceived and designed the experiments: IP, JC-L, HC, MA, and JF. Performed the experiments: IP and JC-L. Analyzed the data: IP, JC-L, RC-M, CP, NK, EV-S, and HC. Contributed reagents/materials/analysis tools: IP, RC-M, CP, EV-S, NK and HC. Wrote, reviewed, and accepted the final version of the paper: IP, JC-L, RC-M, CP, EV-S, NK, HC, MA, and JF.

These errors do not change the scientific conclusions of the article in any way.

The original article has been updated.

## Conflict of Interest Statement

The authors declare that the research was conducted in the absence of any commercial or financial relationships that could be construed as a potential conflict of interest.

