# Supplementary figures and images for: Ankylosing Spondylitis Patients Have Impaired Osteoclast Gene Expression in Circulating Osteoclast Precursors
Source: Front Med (Lausanne). 2017 Jan 27;4:5. doi: 10.3389/fmed.2017.00005 (PMC5269449; doi:10.3389/fmed.2017.00005)

## Slide 1
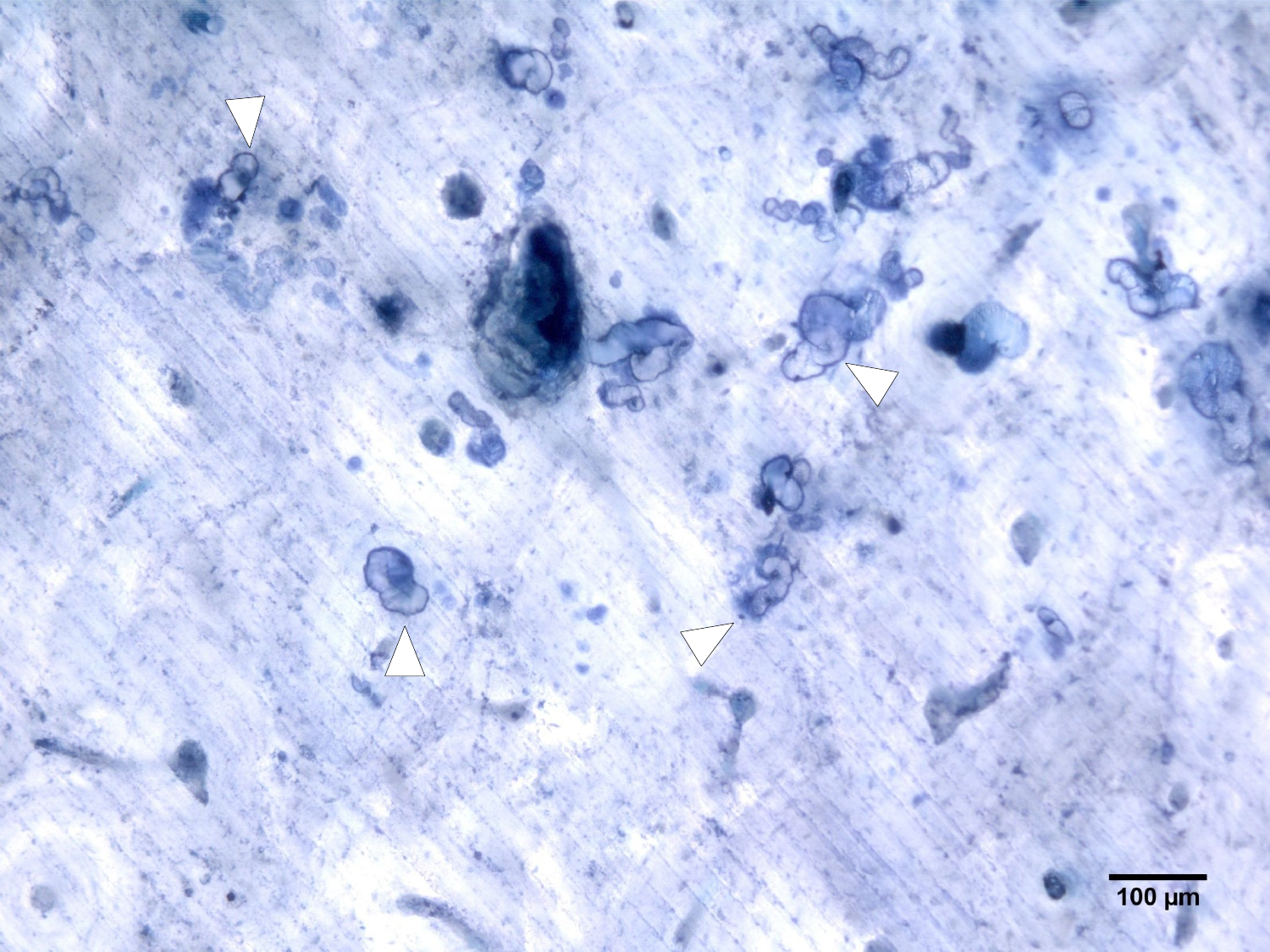

## Slide 2
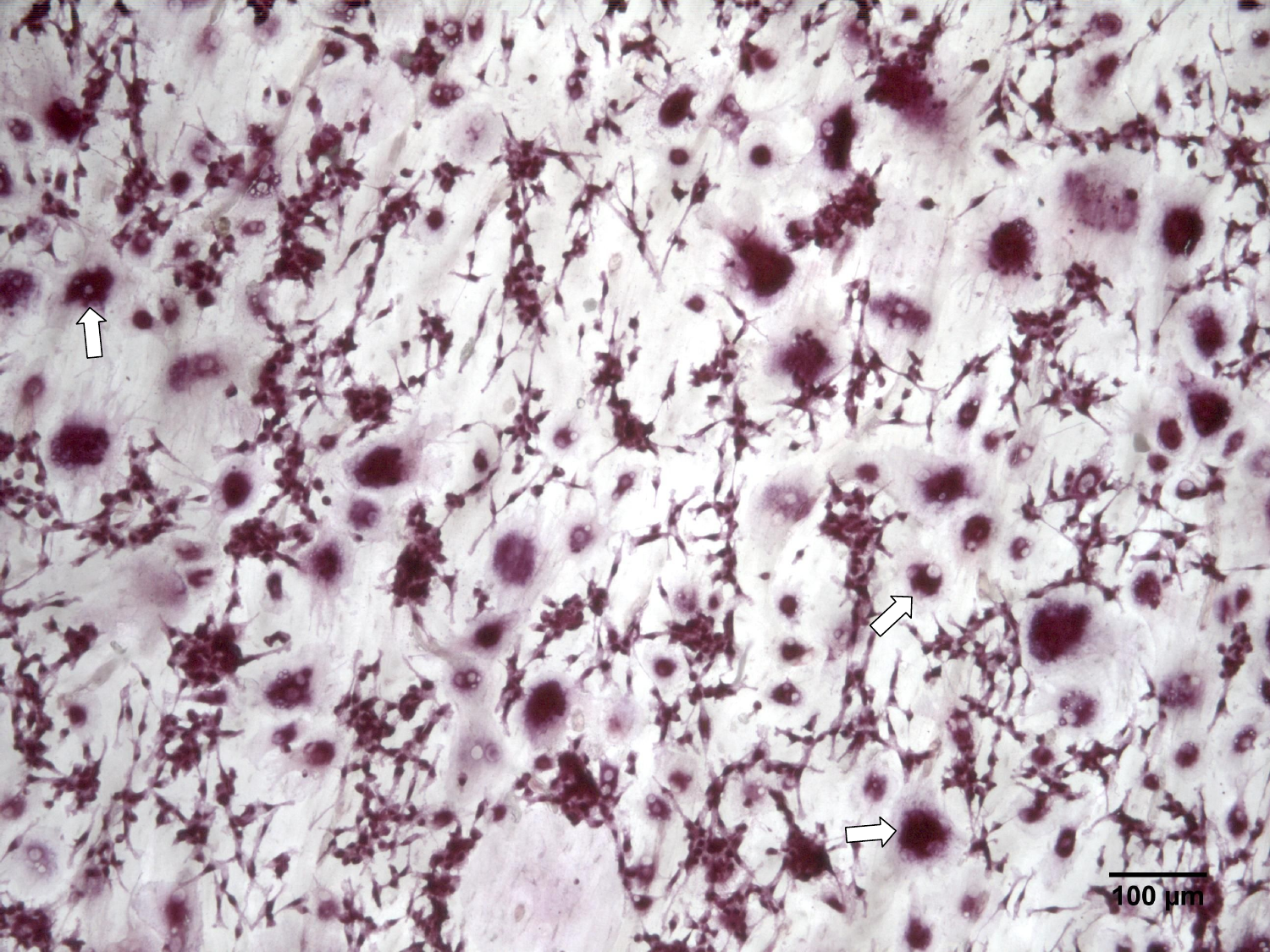

## Slide 3
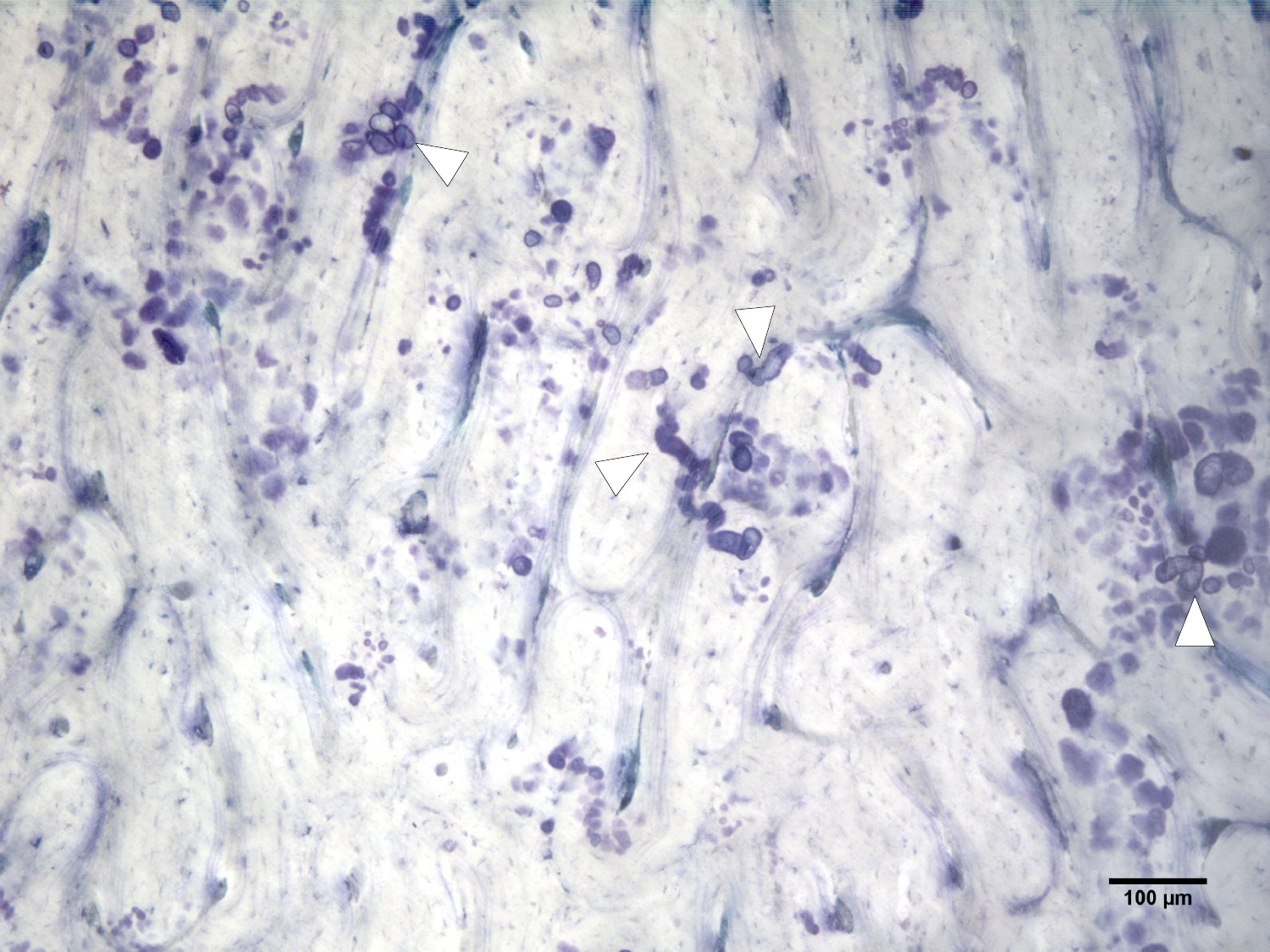

## Slide 4
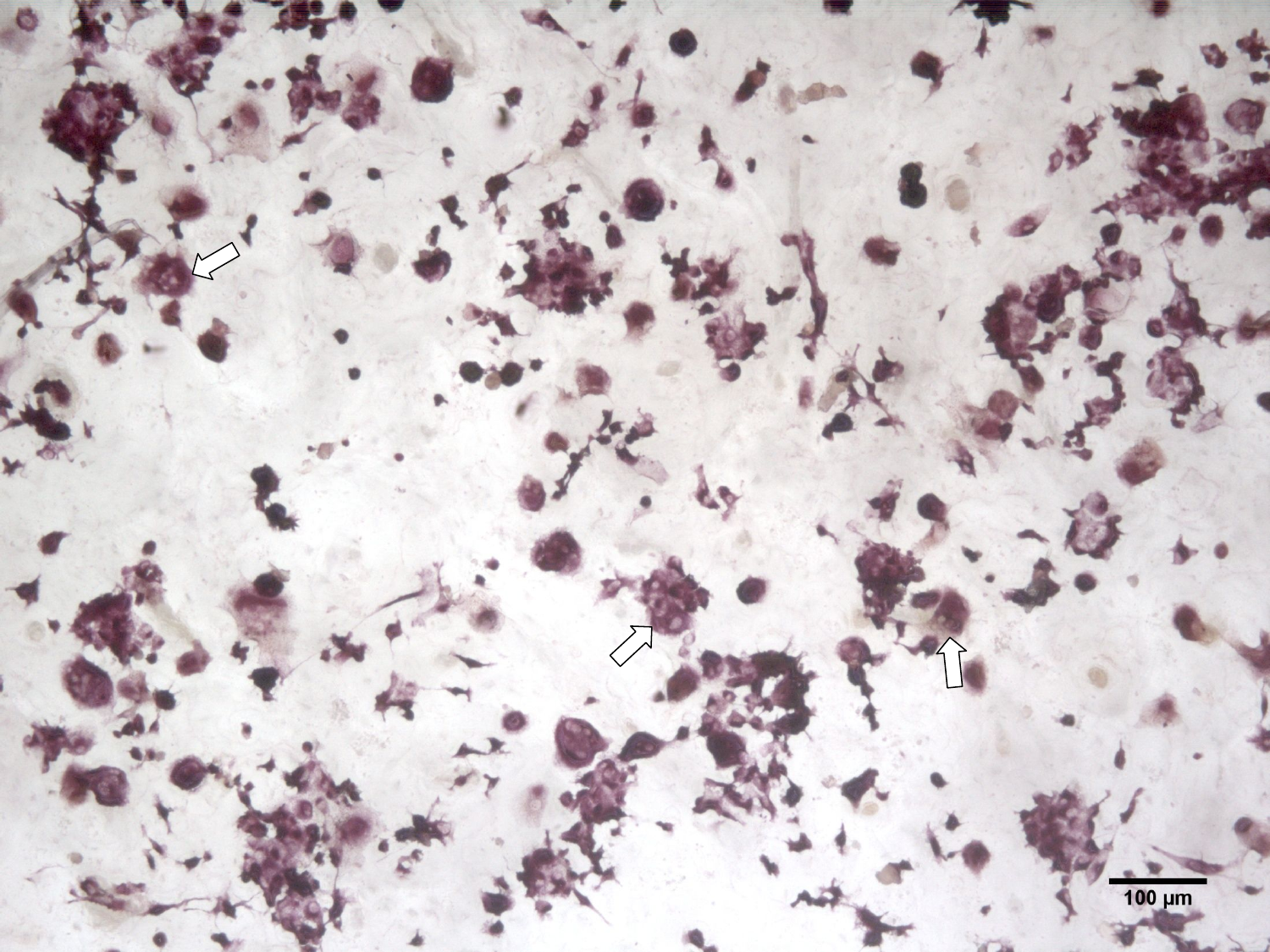

Supplement: Supplementary file 1 [file Presentation_1.PPTX]
